# A systematic literature review of health state utility values in head and neck cancer

**DOI:** 10.1186/s12955-017-0748-z

**Published:** 2017-09-02

**Authors:** Michela Meregaglia, John Cairns

**Affiliations:** 1 Department of Health Services Research and Policy, Faculty of Public Health and Policy, London School of Hygiene and Tropical Medicine, 15-17 Tavistock Place, London, WC1H 9SH UK; 2 CeRGAS (Research Centre on Health and Social Care Management), Bocconi University, Via Roentgen 1, 20136 Milan, Italy; 3 CCBIO (Centre for Cancer Biomarkers), University of Bergen, Postboks 7804, N-5020 Bergen, Norway

**Keywords:** Health state utility values (HSUVs), Health-related quality of life (HRQoL), Head and neck cancer (HNC), Multi-attribute utility instruments (MAUIs), Standard gamble (SG), Time trade-off (TTO), Systematic literature review

## Abstract

**Background:**

Health state utility values (HSUVs) are essential parameters in model-based economic evaluations. This study systematically identifies HSUVs in head and neck cancer and provides guidance for selecting them from a growing body of health-related quality of life studies.

**Methods:**

We systematically reviewed the published literature by searching PubMed, EMBASE and The Cochrane Library using a pre-defined combination of keywords. The Tufts Cost-Effectiveness Analysis Registry and the School of Health and Related Research Health Utilities Database (ScHARRHUD) specifically containing health utilities were also queried, in addition to the Health Economics Research Centre database of mapping studies. Studies were considered for inclusion if reporting original HSUVs assessed using established techniques. The characteristics of each study including country, design, sample size, cancer subsite addressed and demographics of responders were summarized narratively using a data extraction form. Quality scoring and critical appraisal of the included studies were performed based on published recommendations.

**Results:**

Of a total 1048 records identified by the search, 28 studies qualified for data extraction and 346 unique HSUVs were retrieved from them. HSUVs were estimated using direct methods (e.g. standard gamble; *n* = 10 studies), multi-attribute utility instruments (MAUIs; *n* = 13) and mapping techniques (*n* = 3); two studies adopted both direct and indirect approaches. Within the MAUIs, the EuroQol 5-dimension questionnaire (EQ-5D) was the most frequently used (*n* = 11), followed by the Health Utility Index Mark 3 (HUI3; *n* = 2), the 15D (*n* = 2) and the Short Form-Six Dimension (SF-6D; *n* = 1). Different methods and types of responders (i.e. patients, healthy subjects, clinical experts) influenced the magnitude of HSUVs for comparable health states. Only one mapping study developed an original algorithm using head and neck cancer data. The identified studies were considered of intermediate quality.

**Discussion:**

This review provides a dataset of HSUVs systematically retrieved from published studies in head and neck cancer. There is currently a lack of research for some disease phases including recurrent and metastatic cancer, and treatment-related complications. In selecting HSUVs for cost-effectiveness modeling purposes, preference should be given to EQ-5D utility values; however, mapping to EQ-5D is a potentially valuable technique that should be further developed in this cancer population.

**Electronic supplementary material:**

The online version of this article (10.1186/s12955-017-0748-z) contains supplementary material, which is available to authorized users.

## Background

Cost-utility models are increasingly used to establish whether the cost of a new treatment is justified in terms of health gains. This approach usually adopts the quality-adjusted life year (QALY) as a measure of health effectiveness. According to Neumann et al., the QALY corresponds to *the time spent in a series of quality-weighted health states*, where the weights represent the desirability of living in that state [1]. The basic idea is that individuals move through health states over time and that each health state has a preference weight attached to it [2], also known as a health state utility value (HSUV). Thus, the HSUV can be interpreted as the strength of preference for a given health state on a cardinal scale anchored at 0 (‘death’) and 1 (‘full health’), with some instruments also allowing for negative values representing states worse than death [3]. Therefore, QALYs are obtained by summing-up the products of the time spent in each health state and its corresponding preference-based value [4].

HSUVs can be estimated in a variety of ways including direct methods, multi-attribute utility instruments (MAUIs), mapping functions and expert opinion. The most common ways of eliciting HSUVs directly are gambling with respect to a hypothetical treatment that may result in perfect health or death (standard gamble, SG) or trading-off part of future life for a shorter time in perfect health (time trade-off, TTO) [5]. A further, simpler option is to use a visual analog scale (VAS), also known as rating scale, which provides an immediate valuation of the current (or a hypothetical) health state on a graduated scale, usually ranging between 0 and 100. This technique is generally considered to be methodologically inferior to choice tasks such as SG and TTO, which incorporate some extra information about the individual risk attitude [4]; VAS scores, indeed, are elicited in a choice-less context, and thus do not required respondents to make trade-offs within their utility function [6]. Moreover, the rating scales are well-known to present measurement biases such as context bias, spacing-out bias, and end-aversion bias [4, 7]. Additionally, there is now consensus that health-related quality of life (HRQoL) is a multi-dimensional concept, which includes domains related to physical, mental, emotional, and social functioning that are difficult to measure on a single scale [8].

Direct measurement of health utility through SG or TTO can be complicated and time-consuming and lead to incomparable results across the studies due to arbitrary health state descriptions (also called ‘vignettes’) [9, 10]. Consequently, in recent years, HSUVs have been increasingly estimated indirectly using multi-attribute utility instruments (MAUIs). These tools are formed of a generic HRQoL questionnaire and an accompanying formula or set of weights (or “tariffs”) elicited from a sample of the general population for converting responses into HSUVs; thus, the utility measure can be considered as a preference-based evaluation of a given health state described by the dimensions of the tool [11, 12]. The National Institute for Health and Care Excellence (NICE) and the European Network for Health Technology Assessment (EUnetHTA) recommend the EuroQol 5-dimension questionnaire (EQ-5D) (https://euroqol.org) [13, 14]. Accordingly, the TTO with a 10-year time horizon is the most frequently used approach among the direct techniques, because of greater comparability with the method used to develop the EQ-5D scoring algorithm [15]. The other generic MAUIs mostly adopted in the literature [11] are the Health Utility Index (HUI mark 2, HUI2 or mark 3, HUI3) [16], the Short Form-6-dimension (SF-6D) questionnaire derived from the 36-item Short Form Survey (SF-36) (https://www.sheffield.ac.uk/scharr/sections/heds/mvh/sf-6d), the 15D (www.15d-instrument.net), the Quality of Wellbeing (QWB) index [17] and the Assessment of Quality of Life (AQoL) instruments [18].

In many situations, clinical studies neither administer preference-based MAUIs nor elicit HSUVs directly, but collect instead disease-specific HRQoL data or other clinical measures that are not associated with a preference-based scoring system; thus, QALY calculation from these studies is not possible. As a second-best solution, “mapping” or “cross-walking” has been developed to predict HSUVs from non-preference-based scores, provided that a statistical relationship can be established between the two instruments and, sometimes, allowing for the mediating effect of demographic and clinical characteristics [19].

This study focuses on HSUVs in head and neck cancer (HNC). Patients with HNC often undergo several rounds of treatment during which they experience acute toxicity and other side effects, such as loss of verbal abilities, difficulties in swallowing, and considerable pain [20]. This HRQoL impairment may continue long after treatment through persistent functional deficits, physical disfigurement, psychological distress, and recurrent disease. There is an extensive HRQoL literature in HNC, although mainly comprised of disease-specific, non-preference based data unsuitable for cost-utility comparisons. Due to the paucity of HSUVs for some health states in HNC, some previous cost-effectiveness analyses [21, 22] relied on values calculated for other cancers (such as breast or lung) to populate their models with utility parameters. A systematic review published in 2006 [23] identified eight studies providing utility values in HNC elicited through VAS, TTO or SG. Our study extends the collection of utility values related to this medical condition by systematically reviewing the studies published to date. This paper considers for inclusion studies of any design in which utility values in HNC were:

- directly elicited using standard techniques such as TTO or SG either in patient-based studies or in the general population;
- calculated indirectly from patient’s responses to generic MAUIs (e.g. EQ-5D) through a set of tool- and country-specific preference weights;
- predicted from non-preference based HRQoL instruments using mapping algorithms.

The Preferred Reporting Items for Systematic Reviews and Meta-Analyses (PRISMA) statement [24] is not entirely applicable to systematic reviews of HSUVs [25], since the standard Population, Intervention, Comparator, and Outcome (PICO) elements do not provide a useful framework for identifying utility values for health states that are not necessarily attached to a given intervention [26]. Thus, in this study we follow the recommendations provided by Papaioannou et al. [26]. The ultimate objective is to generate a database of HSUVs that might be useful to populate future cost-utility studies of interventions in HNC. In addition, we critically appraise the included studies by highlighting a few elements that should be considered when selecting utility parameters for modeling.

## Methods

**Fig. 1:**
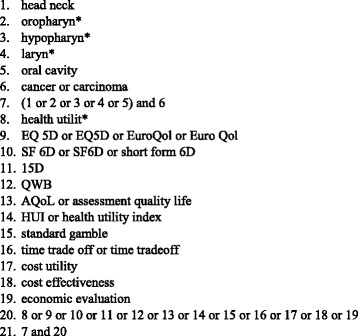
Free-text terms for electronic database searching

A systematic literature search was carried out of the PubMed, EMBASE and Cochrane Library databases for studies published from 2000 until the end of 2016 using a range of free-text terms in title/abstract (Fig. 1). Since Medical Subject Headings (MeSH) terms provide little coverage of HSUVs [25, 26], we identified a few relevant free-text terms by referring to the published recommendations [26] and recent analogous systematic reviews [25, 27, 28]. Tool- (e.g. EQ-5D) and method-specific (e.g. SG) terms were combined with vocabulary related to HNC including the most frequent cancer sites; in using free-text terms, we considered that some instruments may be referred to or spelled in different ways. We did not explicitly included VAS among the keywords, due to the above-mentioned limitations in using this tool for measuring utility. Other search strings were used to identify cost-effectiveness and cost-utility studies using HSUVs to calculate QALYs. We searched directly utility weights in the Tufts Cost-Effectiveness Analysis (CEA) Registry [29] and the University Sheffield School of Health and Related Research Health Utilities Database (ScHARRHUD) [30]. An additional search was carried out of the Health Economics Research Centre (HERC) database [31] to retrieve mapping studies deriving utility values from non-preference based instruments in HNC. We selected the relevant databases based on previous recommendations [26] and systematic reviews on the topic [32]. Web searches of grey literature were not performed to avoid obtaining contents which are frequently subject to changes and cannot be identified in a systematic manner.

All search results were extracted in an Excel spreadsheet and duplicates removed. Titles and abstracts were screened by two independent reviewers and records excluded if not meeting the inclusion criteria; full-text papers were retrieved in case of doubtful results. Articles estimating HNC utility values using established methods were included; studies using the VAS instrument were not considered for inclusion, unless alongside other valuation techniques. This choice is consistent with the suggestion that VAS should be used as an introductory task but not as a definitive method to elicit utility values alone [33]. The included studies had to be published as full-text with no time or language restrictions; conference abstracts, editorials, and reviews were not suitable for inclusion. Studies were excluded if they did not report original utility values in HNC; however, the bibliography of studies referring to secondary sources for HSUVs was checked to avoid missing any relevant publications. The authors resolved any disagreements by discussion until consensus was reached.

The characteristics of the included studies were extracted by the first reviewer (MM) using a form developed following previous studies [25–27], and subsequently crosschecked by the other author (JC). Information collected included: study country, study design, sample size, valuation technique, administration method, cancer subsite addressed, and clinical and demographic characteristics of respondents. For each HSUV, we recorded the number of respondents, the point estimate (i.e. mean or median) and its measure of variance (e.g. standard deviation); the same information was collected for each study subgroup (or time point) whenever applicable.

Although there are no agreed reporting standards for HSUVs studies, the methodological quality of each included study was evaluated through a set of generic criteria as reported by the guidelines from Papaioannou et al. [26]. Thereafter, one point was awarded to each of the following criteria: (1) sample size ≥100; (2) description of respondent selection and recruitment; (3) description of inclusion/exclusion criteria; (4) response rate ≥ 60% [34]; (5) reporting of the amount and reasons of loss to follow-up (only for longitudinal studies); (6) reporting of missing data pattern and methods to deal with it; (7) appropriateness of measure (based on the authors’ judgment). Lastly, the scores were summed for each article to yield an overall quality score, ranging from 0 to 7 where higher scores indicated higher quality [35]. Any other problems arising from the studies (criterion 8) were narratively discussed. Additionally, the International Society for Pharmacoeconomics and Outcomes Research (ISPOR) recently published a set of recommendations for mapping studies [19] that were used to evaluate the quality of mapping studies retrieved by the systematic search.

## Results

### Study selection

**Fig. 2:**
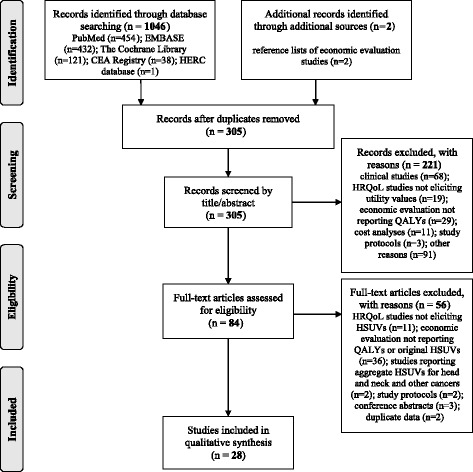
PRISMA flow chart

The PRISMA diagram [24] for this literature search is presented in Fig. 2. In total, the search strategy identified 1048 articles: 1046 were retrieved by searching the online databases (PubMed; EMBASE; The Cochrane Library; CEA Registry; HERC database), and two by manually searching the bibliography of model-based economic evaluations retrieved from the online search. No articles were obtained from the ScHARRHUD database. After removing 743 duplicates, 305 records were scanned for title/abstract and 221 were excluded in this first phase for a variety of reasons reported in the chart. Subsequently, 84 full-text articles were retrieved and a further 56 records were excluded for not complying with the inclusion/exclusion criteria. Accordingly, 28 studies were definitively included in the review.

### Study characteristics

The 28 journal articles included in the review are categorized into three groups: studies using direct elicitation methods (*n* = 10), studies administering MAUIs (*n* = 13) and studies deriving HSUVs using mapping (*n* = 3); two studies [36, 37] adopted both direct methods and MAUIs. The characteristics of the 25 studies using direct and indirect techniques (i.e. MAUIs) are listed in Table 1, while the three mapping studies are separately described in Table 2.

**Table 1:** Studies estimating HSUVs in HNC using direct or indirect methods (*n* = 25)

Author (year)	Country	Study design	Cancer subsite(s)	Valuation method	Mode of administration	Sample size	Response rate	Participants	(Mean) age; % male
Aro (2016) [56]	Finland	Longitudinal	All	15D	Self-completion (by post)	214	72%	Patients	63.0; 66%
Conway (2012) [38]	Australia	Cross-sectional	Oropharynx	SG	1-h group session	99	84%	Healthy subjects	43.0; 54%
de Almeida (2014) [44]	US	Cross-sectional	Oropharynx	SG; VAS	Face-to-face interview	59	NA	Healthy subjects (*n* = 50) and experts (*n* = 9)	Healthy subjects: 34.8; 42%. Experts: 45.3; 89%
del Barco Morillo (2016) [47]	Spain	Longitudinal	All	EQ-5D-3 L	NA	40	NA	Patients	61 (Median); 87%
Govers (2016) [54]	Netherlands	Cross-sectional	Oral cavity	EQ-5D-3 L	Self-completion (by post)	181	62%	Patients	64.4; ≥50%
Hamilton (2016) [39]	UK	Cross-sectional	Larynx	TTO	Face-to-face interview	114	NA	Healthy subjects (*n* = 51) and COPD patients (*n* = 63)	67.3; 49%
Higgins (2011) [58]	Canada	Cross-sectional	Larynx	HUI3	Self-completion	30	NA	Patients	NA
Hollenbeak (2001) [40]	US	Cross-sectional	All	TTO	NA	8	80%	Patients	NA
Jalukar (1998) [41]	US	Cross-sectional	All	TTO	Self-completion (on site for patients; by email for healthcare professionals)	185	Patients: 78%; healthcare professionals: 42%; students: NA	Patients (*n* = 49); healthcare professionals (*n* = 50); students (*n* = 86)	Patients: 57.2; 71% Healthcare professionals: 40.1; 40%; students: NA
Kent (2015) [59]	US	Cross-sectional	Oral cavity and pharynx	SF-6D/VR-6D	Mail or telephone	580	62%	Patients	67.7; 60%
Llewellyn-Thomas (1993) [45]	Canada	Longitudinal	Larynx	TTO/VAS	Interview	66	NA	Patients	NA; 86%
Loimu (2015) [57]	Finland	Longitudinal	Pharynx, larynx, nasal cavity	15D	Self-completion: on site (first assessment); by post (afterwards)	64	76%	Patients	61.6; 75%
Marcellusi (2015) [36]	Italy	Cross-sectional	All	TTO; EQ-5D-3 L	Computer-guided	79	NA	Patients	65.0; 78.5%
Noel (2015) [37]	Canada	Cross-sectional	All	SG; TTO; VAS; EQ-5D-5 L; HUI3	Face-to-face interview	100	79%	Patients	61.0; 75%
Ouattassi (2016) [48]	Morocco	Cross-sectional	All	EQ-5D-3 L	Self-completion	120	NA	Patients	57.0; 60%
Parrilla (2015) [49]	Italy	Longitudinal	Larynx	EQ-5D	Self-completion	30	NA	Patients	68.7; 93%
Pickard (2016) [52]	US	Cross-sectional	All	EQ-5D-3 L	Self-completion	50	NA	Patients	56.0; NA
Pottel (2015) [55]	Belgium	Longitudinal	All	EQ-5D-3 L	Self-completion or interview on site (first assessment); by post (afterwards)	81	81%	Patients	72.0; 86%
Ramaekers (2011) [51]	Netherlands	Cross-sectional	All	EQ-5D-3 L	Self-completion	396	93%	Patients	63.2; 70%
Ringash (2000) [42]	Canada	Cross-sectional	Larynx	TTO	Face-to-face interview	120	49%	Patients	65; 83%
Rogers (2006) [50]	UK	Cross-sectional	Oral cavity and oropharynx	EQ-5D-3 L	Self-completion (by post)	224	64%	Patients	65; 58%
Szabo (2012) [20]	Canada	Cross-sectional	Larynx, lip, oral cavity, oropharynx	SG	Interview using script and prop	101	95%	Healthy subjects	47; 48%
Truong (2016) [53]	US	RCT	Oropharynx, hypopharynx, larynx	EQ-5D-3 L	Self-completion	818	87%	Patients	Arm CIS: 56.1; 86%. Arm CET/CIS: 57.3; 89%
van der Donk (1995) [46]	Netherlands	Cross-sectional	Larynx	TTO/SG/VAS	Face-to-face interview	39	NA	Laryngeal cancer patients (*n* = 10), FOM cancer patients (*n* = 10), experts (*n* = 9), healthy subjects (*n* = 10)	Laryngeal cancer patients: 62; FOM cancer patients: 56; experts: 43; healthy subjects: 36
Weiss (1994) [43]	US	Cross-sectional	Pharynx, larynx	TTO	NA	3	NA	Clinical experts	NA

*Abbreviations: CIS* radiation-cisplatin without cetuximab, *CET/CIS* radiation-cisplatin with cetuximab, *COPD* chronic obstructive pulmonary disease, *EQ-5D-3 L* EuroQol 5-dimension 3-Level, *EQ-5D-5 L* EuroQol 5-dimension 5-Level, *FOM* floor-of-the-mouth, *HNC* head and neck cancer, *HSUV* health state utility value, *HUI3* Health Utility Index Mark 3, *NA* not available, *SF-6D* Short Form-6-dimension, *SG* standard gamble, *TTO* time trade off, *VAS* visual analogue scale, *VR-6D* Veterans RAND-6-dimension

**Table 2:** Mapping studies predicting HSUVs in HNC (*n* = 3)

Author (year)	Country	Mapping technique	From (tool 1)	To(tool 2)	Sample’s description (algorithm)	Sample size (algorithm)	Study ref. (algorithm)	Sample’s description(tool 1)	Sample size (tool 1)	Study ref. (tool 1)
Chan (2014) [60]	Canada	OLS	UW QOL v4	EQ-5D-3 L	Patients treated for HNC	89 (estimation); 48 (validation)				
Parthan (2009) [61]	UK	Published algorithm	EORTC QLQ-C30	EQ-5D-3 L	Patients with liver metastases	75	Krabbe (2004) [63]	Patients with locally advanced inoperable HNC	358	Vermorken (2007) [66]
Yong (2012) [62]	Canada	Published algorithm	SF-36	HUI2	Various patients	6921	Nichol (2001) [64]	Patients with early stage nasopharyngeal cancer	51	Pow (2006) [65]

*Abbreviations: EORTC QLQ-C30* European Organization for Research and Treatment of Cancer Quality of Life Core Questionnaire, *EQ-5D-3 L* EuroQol 5-dimension 3-Level, *HNC* head and neck cancer, *HSUV* health state utility value, *HUI3* Health Utility Index Mark 3, *OLS* ordinary least square regression, *SF-36* 36-Item Short Form Health Survey, *UW QOL v4* University of Washington Quality of Life questionnaire, version 4

#### Studies using direct or indirect methods

Among the studies using direct elicitation techniques, SG alone was adopted in two cases [20, 38] and TTO alone in five [39–43]. In four studies [37, 44–46], more than one direct methodology (i.e. SG, TTO, VAS) was adopted to derive utility values. The study by Noel et al. [37] compared these direct techniques with MAUIs (i.e. EQ-5D, HUI3), while a further study [36] used both TTO and EQ-5D instruments.

In studies administering MAUIs, EQ-5D was the most common (*n* = 11); five of these studies [36, 37, 47–49] did not report which scoring algorithm was used, two studies [50, 51] explicitly adopted the UK algorithm, another two [52, 53] adopted the US one, one study [54] used the Dutch tariff and another one [55] the Belgian one. Moreover, nine of the studies using EQ-5D [36, 47, 48, 50–55] explicitly referred to the 3-level version (EQ-5D-3 L) and one [37] to the newer 5-level one (EQ-5D-5 L); one study [49] did not specify the instrument’s version adopted. Additional generic, preference-based HRQoL tools retrieved by our search were 15D (*n* = 2), HUI3 (*n* = 2) and SF-6D (*n* = 1); no studies used the QWB scale or the AQoL-8D utility instrument.

The 25 articles reported on HNC utility-related studies conducted in several European (Belgium, *n* = 1; Finland, *n* = 2; Italy, *n* = 2; Netherlands, *n* = 3 Spain; *n* = 1 United Kingdom, *n* = 2) and non-European countries (Australia, *n* = 1; Canada, *n* = 5 Morocco, *n* = 1; United States, *n* = 7). The great majority of the HSUVs came from cross-sectional surveys (*n* = 18); the remaining articles (*n* = 7) adopted a longitudinal design, including five prospective cohort studies [45, 49, 55–57] and two clinical trials [47, 53].

Sample sizes varied widely from 3 [43] to 818 [53], with a mean of 152 respondents per study. The response rate was between 49% [42] and 95% [20]. In most of the studies (*n* = 18), the participants were HNC patients at various stages of disease and treatment pathway; in two studies [20, 38] healthy individuals from the general population were surveyed through the SG techniques, while in one case [43] the utility assessment was based on a consultation with a panel of experienced physicians. The remaining four studies [39, 41, 44, 46] retrieved utility measures from multiple subjects (i.e. healthy people, clinical experts, HNC patients and patients with other medical conditions) and reported HSUVs from each group separately.

In studies recruiting HNC patients, most were male and the mean age was always above 55. Conversely, responders were generally younger and with a higher proportion of females in studies surveying individuals from the general population or clinical experts. The range of cancer subsites addressed by each study was quite broad: ten studies [36, 37, 40, 41, 47, 48, 51, 52, 55, 56] generally investigated utility in HNC without specifying any cancer site, six [39, 42, 45, 46, 49, 58] were related to laryngeal cancer, two [38, 44] addressed cancer in the oropharynx, one [54] recruited patients affected by cancer in the oral cavity and the remaining six [20, 43, 50, 53, 57, 59] focused on selected multiple sites (e.g. oropharynx, hypopharynx, and larynx).

The most common way (*n* = 12 [41, 48–58]) of collecting utility data was by self-completion of a written survey (administered on site or by post/e-mail), followed by face-to-face interviews (*n* = 6 [37, 39, 42, 44–46]); four studies adopted different administration options including group session (*n* = 1 [38]), telephone or mail interview (*n* = 1 [59]), interview using a script/prop (*n* = 1 [20]), and computer-guided data collection (*n* = 1 [36]). The administration method was not specified in three cases [40, 43, 47]. When HSUVs were obtained from the patients, the survey (or the interview) was usually scheduled during a clinical appointment or a hospital admission; in longitudinal studies [55, 57], surveys after the first were frequently delivered by post to the patient’s home address.

#### Mapping studies

The three studies deriving HSUVs in HNC using a mapping technique are described in Table 2. Among them, only one [60] developed an original mapping algorithm using responses from HNC patients and was retrieved from the HERC database. Ordinary Least Square (OLS) regression was applied to establish a statistical relationship between the University of Washington Quality of Life questionnaire version 4 (UW QOL v4) and the EQ-5D-3 L using a dataset of 89 patients treated for HNC. Thereafter, the responses of an additional 48 patients enrolled in the study were used as a validation database.

The other two studies [61, 62] were model-based economic evaluations reporting HSUVs for several HNC-related health states by applying previously published algorithms [63, 64] to HRQoL data retrieved from the literature [65, 66].

### Overview of HSUVs

A total of 346 original HSUVs were retrieved from 27 studies included in the review (Additional file 1: Table S1), since one study [41] reported results only graphically in the article. The studies [37, 44, 46] providing the highest number of HSUVs (i.e. over 40) either adopted multiple techniques or interviewed several groups of respondents that yielded different values for each health state. In other cases [45, 49, 53, 55–57, 62], different HSUVs have been collected by the same participants over the study time points. HSUVs were reported as means in the great majority of studies (*n* = 25), of which four [20, 38, 51, 53] also reported the median; the remaining two studies [47, 55] calculated a median value only. Among the measures of variance, standard deviation was the most frequently adopted (*n* = 12), followed by the min-max range (*n* = 7), and the interquartile range (*n* = 5); several studies reported more than one measure type. In some cases [39, 43, 46, 58, 62], no measures of variability were reported, thus limiting the usefulness of the utility data.

### Study quality assessment

The quality assessment of the 25 studies using direct or indirect methods was based upon eight criteria, of which seven were given a score (Table 3). In all studies, the instrument adopted to estimate HSUVs was considered appropriate in relation with the participants enrolled. Additionally, most studies (84%) reported a description of the participants recruitment process, whilst only 56% of them clearly stated the inclusion/exclusion criteria. Information on missing data and techniques to deal with them were reported by a limited number of studies (24%). In half of the studies, the sample size was rather small (<100) and response rate was either low (<60%) or not reported. In reviewing these studies, we highlighted a few additional issues (criterion 8 [26]) that should be considered when selecting sources to populate health economic models with utility parameters. First, some of the included studies are quite dated (published before 2000), thus describing health states that might not be realistic nowadays because of emerging treatment modalities, improvements in treatment-related morbidity and organ preservation techniques. Second, there might be potential sources of bias in reporting HRQoL results in clinical studies investigating one or more interventions, although the number of comparative trials retrieved by our search was very limited. Third, in some studies [36, 48, 59, 60], and especially those analyzing HRQoL in multiple cancers including head and neck [36, 59], patient’s characteristics (e.g. cancer stage/site, treatment phase) are poorly reported, thus making it difficult to match the study’s HSUVs with the health states described in a cost-effectiveness model. Lastly, the great majority of studies are cross-sectional surveys, representing the quickest and cheapest method for gathering HRQoL data; however, longitudinal data collections are often more valuable since they facilitate capture of changes in utility values as cancer progresses through different phases.

**Table 3:** Quality assessment criteria (from Papaioannou et al. 2013) in studies using direct or indirect methods (*n* = 25)

Criteria	[56]	[38]	[44]	[47]	[54]	[39]	[58]	[40]	[41]	[59]	[45]	[57]	[36]	[37]	[48]	[49]	[52]	[55]	[51]	[42]	[50]	[20]	[53]	[46]	[43]	Tot
Sample size	1	0	0	0	1	1	0	0	1	1	0	0	0	1	1	0	0	0	1	1	1	1	1	0	0	12/25
Selection and recruitment	1	1	1	1	1	1	0	0	1	1	1	1	1	1	1	1	1	1	1	1	1	1	0	1	0	21/25
Inclusion/ exclusion criteria	0	1	1	1	0	1	0	0	1	0	1	0	1	1	0	1	0	1	1	1	0	1	0	1	0	14/25
Response rate^a^	1	1	0	0	1	0	0	1	0	1	0	1	0	1	0	0	0	1	1	0	1	1	1	0	0	12/25
Loss to follow-up^b^	1	C	C	0	C	C	C	C	C	C	0	1	C	C	C	1	C	1	C	C	C	C	1	C	C	5/7
Missing data	0	0	0	0	0	0	0	0	0	1	0	1	0	0	0	0	0	1	1	0	0	0	1	1	0	6/25
Appropriateness of measure	1	1	1	1	1	1	1	1	1	1	1	1	1	1	1	1	1	1	1	1	1	1	1	1	1	25/25
Total score	5/7	4/6	3/6	3/7	4/6	4/6	1/6	2/6	4/6	5/6	3/7	5/7	3/6	5/6	3/6	4/7	2/6	6/7	6/6	4/6	4/6	5/6	5/7	4/6	1/6	

^a^Set equal to 0 if <60% or not reported in the study

^b^C: cross-sectional study (not applicable criterion)

With reference to mapping, we retrieved only one original algorithm [60] through the search, thus preventing a comparative evaluation of studies. This study presented a four-variable model to predict EQ-5D-3 L utilities using OLS regression; coefficient values and error terms were clearly reported and box-plot distributions of actual and predicted utilities provided. However, the authors did not justify the model choice in relation to the observed EQ-5D distribution, nor any additional tests or judgments made. The goodness-of-fit was presented as R,^2^ mean absolute error (MAE) and root mean squared error (RMSE), which are considered of limited value in the mapping field. No demographic or clinical variables were included as covariates, which was recognized as a study limitation by the same authors. Moreover, when the sample size is small (as it was in this study), the most recent guidelines do not recommend splitting it for empirical validation [19].

## Discussion

This study reviews systematically published studies reporting HSUVs in HNC. Compared to a previous review [23], many more studies have been identified, most of which use the EQ-5D and were published from 2011 onwards. Overall, this review shows that HNC patients suffer from substantial HRQoL impairment over the different disease phases. However, there is a lack of research into the HRQoL in the recurrent and/or metastatic health states, with only one study [47] reporting a median EQ-5D utility value (i.e. 0.7) from the patients, which is less useful for the purposes of economic evaluation that focuses on mean costs and effects. Another study [44] elicits values for a range of recurrent disease states from healthy subjects and clinical experts using SG and VAS and obtains extremely heterogeneous results across the types of participants and methods. The same paucity of HSUVs was observed for treatment-related complications, which are addressed by three studies [20, 44, 45] only, possibly because of the infrequency of some of these events that restricts the data from patients in that health state.

Differences in utilities were found across studies even in the pre-treatment state. The choice of baseline utility is particularly relevant because it affects the incremental gain achievable by different therapeutic options [13], thus potentially biasing the estimated cost-effectiveness. The two Finnish studies [56, 57] using the 15D yielded higher utility values in patients shortly after diagnosis than those using the EQ-5D [53, 54]. This phenomenon has previously been observed in studies addressing other medical conditions [32, 67, 68]. There are many possible explanations for these discrepancies: different number of dimensions, the EQ-5D has generally been valued using TTO rather than VAS [69], the preference weights have come from different populations (a Finnish value set is usually adopted for 15D) [67], and the EQ-5D, unlike the 15D, can take negative values [69, 70]. The participants’ characteristics might have also affected study results. For example, a study [55] addressing HRQoL in patients aged ≥65 years with HNC consistently provided lower HSUVs than other studies in either the pre-treatment, treatment, and follow-up phases, probably because of comorbidities and functional impairments usually affecting elderly people independently from cancer. Moreover, the use of different scoring algorithms may have contributed to variation in HSUVs in studies administering the EQ-5D.

Heterogeneity in utility values was particularly evident in the studies applying more than one technique to evaluate the same health state. Among them, in a study reporting HSUVs for different treatments, treatment-related complications, and remission/recurrence states in oropharyngeal cancer [44], the values obtained using a VAS scale were consistently lower that for the SG. In the study by Marcellusi et al. [36], patients in follow-up after treatment for HNC reported lower utility values when performing the TTO task than when responding to the EQ-5D questionnaire. Another study [37] compared five different (direct and indirect) methods to retrieve HSUVs from patients experiencing a similar health state (i.e. three months after completion of treatments and no evidence of recurrent disease). Unlike Marcellusi et al. [36], the method yielding the highest utility value in the overall sample (*n* = 100) was TTO (0.94), followed by SG (0.91), EQ-5D (0.82), VAS (0.76) and HUI3 (0.75). That VAS scores are consistently lower than SG scores is well-known in the literature; in 2001, Torrance et al. [33], after reviewing several studies, concluded that the relationship between the two instruments can be represented by a concave curve passing through 0 and 1. Moreover, the indirect methods involving MAUIs have been shown to yield systematically lower utility values than the direct ones in a wide range of diseases [71] for a variety of reasons. First, in MAUIs participants are not asked to consider their health status relative to death and thus, there is no disincentive in reporting more severe health problems [72]. Second, respondents are forced to describe their complex medical conditions through a limited number of attributes, thus ignoring any positive feelings that would boost utility values. Third, it is likely that the general population used to obtain tariffs for MAUIs make a different trade-off between a given health state and death because they tend to be younger and healthier. Finally, the vignettes described in direct valuation tasks are usually more detailed than the MAUI health states [71]. In studies comparing alternative MAUIs, EQ-5D has been shown to provide higher utilities values compared to HUI2 and HUI3, which in turn yield higher values than SF-6D. As for the differences between EQ-5D and 15D, potential explanations are likely to be found in descriptive systems, preference measurement, source of community preferences, and scoring methods [73].

In addition, studies can be classified by the type of responders who valued the health states, either patients or healthy subjects. In the literature, some argue that patients are best placed to value the relevant health states, while others advocate valuation by healthy people who will not directly benefit from a new treatment but, in tax-based systems, will bear its cost. The latter claim that this will provide an unbiased estimate of the hypothetical health states [7, 74] and more consistency across appraisal of very different interventions. The review by Komatsuzaki et al. [23] showed that patients usually reported lower utilities than physicians and healthy people for health states associated to HNC. In this review, only a few studies recruited participants from the general population, thus limiting the number of utilities comparisons across different types of responders. One study [46] confirms the conclusions reached by the previous review [23], whilst others [41, 44] found healthy subjects consistently providing lower utility estimates compared to patients and healthcare professionals.

This study facilitates the identification of HSUVs for use in future HNC economic evaluations. The number of retrieved studies was quite large, with almost 350 distinct HSUVs collected from them. Most of the utility values were collected during the treatment phase or shortly after the completion of treatment, whilst limited evidence is available for the health-related utility assessment in HNC recurrent and end-of-life states. Due to the variety of health state definitions and valuation techniques across the studies, we were not able to perform a quantitative synthesis of the results [3]. Moreover, unlike cost-effectiveness studies where structured guidelines exist to support authors and reviewers in assessing their quality [75, 76], recommendations for valuation studies specifically aimed at measuring HSUVs are more fragmented or method-specific [10]. In this review, the assessment of study quality was based on a set of generic recommendations elaborated by a previous study [26] and arbitrarily modified to allow a quantitative scoring of the studies adopting direct and indirect techniques to estimate HSUVs; for mapping studies, we relied instead on recent ISPOR guidelines [19].

Although there is no universally accepted theoretical basis for choosing direct or indirect methods [71], the use of the EQ-5D, is favored by several agencies including NICE, the Canadian Agency for Drugs and Technologies in Health and the French National Authority for Health [3]. In a recent position statement [77], NICE recommends the use of EQ-5D-3 L for base-case analyses, or mapping EQ-5D-5 L responses onto the 3 L valuation set, to derive HSUVs, since further research is needed to explore the impact of adopting the EQ-5D-5 L valuation set on technology appraisal. In model-based cost-effectiveness studies, where there is a choice of HSUVs, those using the value set of the jurisdiction for which a decision is being made are usually preferred. Moreover, HSUVs should be collected from studies enrolling patients with demographic and clinical characteristics that mostly resemble those of potential recipients of the intervention under investigation in the model. Until now, studies relying on direct techniques represent the only available source to retrieve HSUVs for recurrent disease, palliative states, or treatment-related complications in HNC. Although considered as qualitatively inferior to MAUIs [3], these methods can provide values for cost-effectiveness analyses where the ‘vignettes’ presented in the choice task fit with the health states addressed in the model. Finally, in the absence of preference-based data, mapping from disease-specific instruments to generic MAUIs may represent a valuable alternative [74]; however, the only algorithm published to date in HNC [60] does not map from one of the HRQoL tools most frequently adopted in cancer studies, such as the European Organization for Research and Treatment of Cancer 30-item Quality of Life Core Questionnaire (EORTC QLQ-C30 [78]) and the Functional Assessment of Cancer Therapy - General (FACT-G [79]). Greater availability of mapping functions would facilitate the comparison of treatments using HRQoL data from many randomized controlled trials that only collected disease-specific health status information. Overall, the use of different techniques for utility elicitation might have substantial implications in cost-utility analyses; for example, it has been shown [71] that MAUIs, compared to direct valuation, tend to favor non-lifesaving treatments over interventions preventing or delaying death. Thus, regulatory bodies should avoid a mixture of methods in their decision processes to avoid a biased allocation of healthcare resources. Moreover, health economic modelers are always recommended to extensively test the uncertainty around the utility parameters in sensitivity analyses [71].

## Conclusions

This study improves understanding of preference-based HRQoL measurement in HNC by systematically reviewing and critically evaluating studies that estimated HSUVs in this cancer setting. Utility values are an essential parameter but also a major source of uncertainty in model-based economic evaluations, where it is common to select them from a single study based on clinical considerations [3, 28]. Further studies on the health-related utility assessment from HNC patients using MAUIs in recurrent and terminal states are encouraged. Additional research on mapping algorithms to convert disease-specific HRQoL results onto preference-based HSUVs would be of value in this cancer population. Overall, the methods used to identify utility values within a growing body of HRQoL literature should be increasingly systematic and justified in future studies.

## Additional file

Additional file 1: Table S1. Details of HSUVs reported by the studies (*n* = 27). (DOCX 107 kb)
