# Supplementary material for: A systematic literature review of health state utility values in head and neck cancer
Source: Health Qual Life Outcomes. 2017 Sep 2;15:174. doi: 10.1186/s12955-017-0748-z (PMC5581467; doi:10.1186/s12955-017-0748-z)
Supplement: Additional file 1: Table S1. — Details of HSUVs reported by the studies (n = 27). (DOCX 107 kb) [file 12955_2017_748_MOESM1_ESM.docx]

**Table A1.** Details of HSUVs reported by the studies (n=27).

| **Author (year)** | **Method** | **Patient’s characteristics/**  **Health state description** | | | **Group/Time point** | | **N** | **Mean** | **SD** | **SE** | **95% CI** | **Median** | **Range** | **IQR** |
| --- | --- | --- | --- | --- | --- | --- | --- | --- | --- | --- | --- | --- | --- | --- |
| Aro (2016) [56] | 15D | Patients receiving treatment (i.e. surgery, CRT, or combined modality treatment) and followed-up for at least 12 months | | | Baseline | | 214 | 0.872 |  |  |  |  |  |  |
|  |  |  |  |  | 3 months | All | 198 | 0.839 | 0.114 |  |  |  |  |  |
|  |  |  |  |  |  | no PEG | 109 | 0.862 |  |  |  |  |  |  |
|  |  |  |  |  |  | PEG | 88 | 0.810 |  |  |  |  |  |  |
|  |  |  |  |  | 6 months | | 202 | 0.857 |  |  |  |  |  |  |
|  |  |  |  |  | 12 months | | 214 | 0.852 |  |  |  |  |  |  |
| Chan (2014) [60] | Mapping | Patients after treatment for HNC | | | Estimation sample | Actual | 89 | 0.821 |  | 0.03 |  |  |  |  |
|  |  |  |  |  |  | Predicted |  | 0.821 |  | 0.02 |  |  |  |  |
|  |  |  |  |  | Validation sample | Actual | 48 | 0.801 |  | 0.02 |  |  |  |  |
|  |  |  |  |  |  | Predicted |  | 0.791 |  | 0.01 |  |  |  |  |
| Conway (2012) [38] | SG | Oropharyngeal cancer stages II-III treated with ND and CRT/RT and/or surgery | | |  | | 99 | 0.58 |  |  | 0.53-0.63 | 0.65 |  | 0.45-0.75 |
| de Almeida (2014) [44] | VAS | Scenarios describing treatment modalities for oropharyngeal cancer | TORS | | Healthy subjects | | 50 | 0.67 |  |  | 0.61-0.73 |  |  |  |
|  |  |  |  |  | Experts | | 9 | 0.82 |  |  | 0.75-0.89 |  |  |  |
|  |  |  | TORS + adjuvant XRT | | Healthy subjects | | 50 | 0.59 |  |  | 0.62-0.64 |  |  |  |
|  |  |  |  |  | Experts | | 9 | 0.60 |  |  | 0.48-0.72 |  |  |  |
|  |  |  | TORS + adjuvant CRT | | Healthy subjects | | 50 | 0.53 |  |  | 0.47-0.58 |  |  |  |
|  |  |  |  |  | Experts | | 9 | 0.45 |  |  | 0.33-0.57 |  |  |  |
|  |  |  | XRT | | Healthy subjects | | 50 | 0.54 |  |  | 0.49-0.60 |  |  |  |
|  |  |  |  |  | Experts | | 9 | 0.59 |  |  | 0.48-0.70 |  |  |  |
|  |  |  | CRT | | Healthy subjects | | 50 | 0.48 |  |  | 0.43-0.54 |  |  |  |
|  |  |  |  |  | Experts | | 9 | 0.42 |  |  | 0.29-0.54 |  |  |  |
|  | SG |  | TORS | | Healthy subjects | | 50 | 0.95 |  |  | 0.94-0.97 |  |  |  |
|  |  |  |  |  | Experts | | 9 | 0.99 |  |  | 0.97-1.00 |  |  |  |
|  |  |  | TORS + adjuvant XRT | | Healthy subjects | | 50 | 0.89 |  |  | 0.85-0.93 |  |  |  |
|  |  |  |  |  | Experts | | 9 | 0.97 |  |  | 0.94-1.00 |  |  |  |
|  |  |  | TORS + adjuvant CRT | | Healthy subjects | | 50 | 0.89 |  |  | 0.85-0.93 |  |  |  |
|  |  |  |  |  | Experts | | 9 | 0.94 |  |  | 0.91-0.97 |  |  |  |
|  |  |  | XRT | | Healthy subjects | | 50 | 0.91 |  |  | 0.87-0.94 |  |  |  |
|  |  |  |  |  | Experts | | 9 | 0.97 |  |  | 0.94-1.00 |  |  |  |
|  |  |  | CRT | | Healthy subjects | | 50 | 0.88 |  |  | 0.83-0.92 |  |  |  |
|  |  |  |  |  | Experts | | 9 | 0.93 |  |  | 0.88-0.97 |  |  |  |
|  | VAS | Scenarios describing treatment-related complications | Temporary tracheostomy | | Healthy subjects | | 50 | 0.61 |  |  | 0.56-0.66 |  |  |  |
|  |  |  |  |  | Experts | | 9 | 0.53 |  |  | 0.44-0.62 |  |  |  |
|  |  |  | Permanent tracheostomy | | Healthy subjects | | 50 | 0.44 |  |  | 0.38-0.52 |  |  |  |
|  |  |  |  |  | Experts | | 9 | - |  |  | - |  |  |  |
|  |  |  | Temporary gastrostomy | | Healthy subjects | | 50 | 0.54 |  |  | 0.50-0.59 |  |  |  |
|  |  |  |  |  | Experts | | 9 | 0.46 |  |  | 0.29-0.62 |  |  |  |
|  |  |  | Permanent gastrostomy | | Healthy subjects | | 50 | 0.36 |  |  | 0.29-0.43 |  |  |  |
|  |  |  |  |  | Experts | | 9 | - |  |  | - |  |  |  |
|  |  |  | Pharyngocutaneous fistula | | Healthy subjects | | 50 | 0.53 |  |  | 0.47-0.60 |  |  |  |
|  |  |  |  |  | Experts | | 9 | 0.46 |  |  | 0.36-0.57 |  |  |  |
|  |  |  | Febrile neutropenia | | Healthy subjects | | 50 | 0.70 |  |  | 0.65-0.75 |  |  |  |
|  |  |  |  |  | Experts | | 9 | 0.77 |  |  | 0.63-0.91 |  |  |  |
|  |  |  | Esophageal stenosis | | Healthy subjects | | 50 | 0.40 |  |  | 0.35-0.46 |  |  |  |
|  |  |  |  |  | Experts | | 9 | 0.38 |  |  | 0.22-0.53 |  |  |  |
|  |  |  | Osteoradionecrosis | | Healthy subjects | | 50 | 0.41 |  |  | 0.35-0.47 |  |  |  |
|  |  |  |  |  | Experts | | 9 | 0.44 |  |  | 0.33-0.55 |  |  |  |
|  | SG |  | Temporary tracheostomy | | Healthy subjects | | 50 | 0.94 |  |  | 0.92-0.97 |  |  |  |
|  |  |  |  |  | Experts | | 9 | 0.98 |  |  | 0.96-0.99 |  |  |  |
|  |  |  | Permanent tracheostomy | | Healthy subjects | | 50 | 0.85 |  |  | 0.80-0.91 |  |  |  |
|  |  |  |  |  | Experts | | 9 | - |  |  | - |  |  |  |
|  |  |  | Temporary gastrostomy | | Healthy subjects | | 50 | 0.89 |  |  | 0.85-0.94 |  |  |  |
|  |  |  |  |  | Experts | | 9 | 0.98 |  |  | 0.96-0.99 |  |  |  |
|  |  |  | Permanent gastrostomy | | Healthy subjects | | 50 | 0.81 |  |  | 0.74-0.88 |  |  |  |
|  |  |  |  |  | Experts | | 9 | - |  |  | - |  |  |  |
|  |  |  | Pharyngocutaneous fistula | | Healthy subjects | | 50 | 0.89 |  |  | 0.85-0.94 |  |  |  |
|  |  |  |  |  | Experts | | 9 | 0.96 |  |  | 0.92-0.99 |  |  |  |
|  |  |  | Febrile neutropenia | | Healthy subjects | | 50 | 0.96 |  |  | 0.94-0.98 |  |  |  |
|  |  |  |  |  | Experts | | 9 | 0.99 |  |  | 0.98-1.00 |  |  |  |
|  |  |  | Esophageal stenosis | | Healthy subjects | | 50 | 0.85 |  |  | 0.80-0.90 |  |  |  |
|  |  |  |  |  | Experts | | 9 | 0.96 |  |  | 0.94-0.98 |  |  |  |
|  |  |  | Osteoradionecrosis | | Healthy subjects | | 50 | 0.85 |  |  | 0.81-0.90 |  |  |  |
|  |  |  |  |  | Experts | | 9 | 0.96 |  |  | 0.93-0.99 |  |  |  |
|  | VAS | Scenarios describing remission and recurrence | Remission (after TORS) | | Healthy subjects | | 50 | 0.80 |  |  | 0.76-0.85 |  |  |  |
|  |  |  |  |  | Experts | | 9 | 0.87 |  |  | 0.81-0.94 |  |  |  |
|  |  |  | Remission (after TORS/adjuvant XRT or after XRT) | | Healthy subjects | | 50 | 0.75 |  |  | 0.70-0.79 |  |  |  |
|  |  |  |  |  | Experts | | 9 | 0.80 |  |  | 0.75-0.85 |  |  |  |
|  |  |  | Remission (after TORS/adjuvant CRT or after CRT) | | Healthy subjects | | 50 | 0.72 |  |  | 0.67-0.77 |  |  |  |
|  |  |  |  |  | Experts | | 9 | 0.68 |  |  | 0.55-0.80 |  |  |  |
|  |  |  | Local recurrence (requiring surgery) | | Healthy subjects | | 50 | 0.39 |  |  | 0.33-0.45 |  |  |  |
|  |  |  |  |  | Experts | | 9 | 0.45 |  |  | 0.29-0.62 |  |  |  |
|  |  |  | Local recurrence (requiring XRT) | | Healthy subjects | | 50 | 0.51 |  |  | 0.45-0.56 |  |  |  |
|  |  |  |  |  | Experts | | 9 | 0.41 |  |  | 0.27-0.55 |  |  |  |
|  |  |  | Regional recurrence (ND) | | Healthy subjects | | 50 | 0.68 |  |  | 0.62-0.74 |  |  |  |
|  |  |  |  |  | Experts | | 9 | 0.63 |  |  | 0.48-0.79 |  |  |  |
|  |  |  | Distant recurrence (CT) | | Healthy subjects | | 50 | 0.20 |  |  | 0.16-0.24 |  |  |  |
|  |  |  |  |  | Experts | | 9 | 0.18 |  |  | 0.12-0.24 |  |  |  |
|  |  |  | Terminal/palliative state (CT) | | Healthy subjects | | 50 | 0.14 |  |  | 0.10-0.18 |  |  |  |
|  |  |  |  |  | Experts | | 9 | 0.08 |  |  | 0.05-0.11 |  |  |  |
|  | SG |  | Remission (after TORS) | | Healthy subjects | | 50 | 0.96 |  |  | 0.94-0.98 |  |  |  |
|  |  |  |  |  | Experts | | 9 | 0.99 |  |  | 0.98-1.00 |  |  |  |
|  |  |  | Remission (after TORS/adjuvant XRT or XRT) | | Healthy subjects | | 50 | 0.95 |  |  | 0.93-0.98 |  |  |  |
|  |  |  |  |  | Experts | | 9 | 0.98 |  |  | 0.96-1.00 |  |  |  |
|  |  |  | Remission (after TORS/adjuvant CRT or CRT) | | Healthy subjects | | 50 | 0.95 |  |  | 0.92-0.98 |  |  |  |
|  |  |  |  |  | Experts | | 9 | 0.97 |  |  | 0.94-0.99 |  |  |  |
|  |  |  | Local recurrence (surgery) | | Healthy subjects | | 50 | 0.82 |  |  | 0.77-0.87 |  |  |  |
|  |  |  |  |  | Experts | | 9 | 0.92 |  |  | 0.87-0.97 |  |  |  |
|  |  |  | Local recurrence (XRT) | | Healthy subjects | | 50 | 0.88 |  |  | 0.84-0.91 |  |  |  |
|  |  |  |  |  | Experts | | 9 | 0.91 |  |  | 0.87-0.95 |  |  |  |
|  |  |  | Regional recurrence (ND) | | Healthy subjects | | 50 | 0.94 |  |  | 0.91-0.97 |  |  |  |
|  |  |  |  |  | Experts | | 9 | 0.97 |  |  | 0.94-0.99 |  |  |  |
|  |  |  | Distant recurrence (CT) | | Healthy subjects | | 50 | 0.57 |  |  | 0.50-0.64 |  |  |  |
|  |  |  |  |  | Experts | | 9 | 0.43 |  |  | 0.22-0.64 |  |  |  |
|  |  |  | Terminal/palliative state (CT) | | Healthy subjects | | 50 | 0.42 |  |  | 0.34-0.50 |  |  |  |
|  |  |  |  |  | Experts | | 9 | 0.31 |  |  | 0.11-0.51 |  |  |  |
| del Barco Morillo (2016) [47] | EQ-5D | Palliative CT for recurrent or metastatic HNC (untreatable by surgery or re-irradiation) | | | Across all visits (every 8 weeks) | | 40 |  |  |  |  | 0.7 | 0.6-0.8 |  |
| Govers (2016) [54] | EQ-5D (Dutch tariff) | Early stage (I-II) oral cavity cancer patient undergoing different diagnostic and treatment interventions | WW | | All patients | | 26 | 0.804 |  | 0.04 |  |  |  |  |
|  |  |  |  |  | Group 1 | | 21 | 0.849 |  | 0.05 |  |  |  |  |
|  |  |  |  |  | Group 2 | | 20 | 0.826 |  | 0.05 |  |  |  |  |
|  |  |  | SLNB | | All patients | | 19 | 0.863 |  | 0.05 |  |  |  |  |
|  |  |  |  |  | Group 1 | | 18 | 0.859 |  | 0.05 |  |  |  |  |
|  |  |  |  |  | Group 2 | | 18 | 0.858 |  | 0.05 |  |  |  |  |
|  |  |  | SOHND | | All patients | | 104 | 0.834 |  | 0.02 |  |  |  |  |
|  |  |  |  |  | Group 1 | | 86 | 0.841 |  | 0.02 |  |  |  |  |
|  |  |  |  |  | Group 2 | | 53 | 0.849 |  | 0.03 |  |  |  |  |
|  |  |  | MRND | | All patients | | 25 | 0.794 |  | 0.04 |  |  |  |  |
|  |  |  |  |  | Group 1 | | 20 | 0.800 |  | 0.05 |  |  |  |  |
|  |  |  |  |  | Group 2 | |  | - |  | - |  |  |  |  |
| Hamilton (2016) [39] | TTO | Four vignettes describing the treatment process and outcome for advanced laryngeal cancer | CRT, optimal outcome | | All participants | | 114 | 0.64 |  |  |  |  |  |  |
|  |  |  |  |  | Group 1 | | 71 | 0.70 |  |  |  |  |  |  |
|  |  |  |  |  | Group 2 | | 43 | 0.53 |  |  |  |  |  |  |
|  |  |  | CRT, outcome with complications | | All participants | | 114 | 0.31 |  |  |  |  |  |  |
|  |  |  |  |  | Group 1 | | 71 | 0.37 |  |  |  |  |  |  |
|  |  |  |  |  | Group 2 | | 43 | 0.22 |  |  |  |  |  |  |
|  |  |  | TL, optimal outcome | | All participants | | 114 | 0.57 |  |  |  |  |  |  |
|  |  |  |  |  | Group 1 | | 71 | 0.55 |  |  |  |  |  |  |
|  |  |  |  |  | Group 2 | | 43 | 0.59 |  |  |  |  |  |  |
|  |  |  | TL, outcome with complications | | All participants | | 114 | 0.33 |  |  |  |  |  |  |
|  |  |  |  |  | Group 1 | | 71 | 0.34 |  |  |  |  |  |  |
|  |  |  |  |  | Group 2 | | 43 | 0.22 |  |  |  |  |  |  |
| Higgins (2011) [58] | HUI3 (health state A); adjustments of A score (other health states) | Patients with complete response to treatment (XRT/CO2) and no evidence of active disease (health state A) | A: alive with voice box entirely intact | |  | | 30 | 0.8718 |  |  |  |  |  |  |
|  |  |  | B: alive with part of the box intact | |  |  | - | 0.706 |  |  |  |  |  |  |
|  |  |  | C: dead of disease | |  |  | - | 0 |  |  |  |  |  |  |
|  |  |  | D: alive with recurrent/active disease | |  |  | - | 0.307 |  |  |  |  |  |  |
|  |  |  | E: alive without voice box/TL | |  |  | - | 0.366 |  |  |  |  |  |  |
| Hollenbeak (2001) [40] | TTO | Surgical patients | Modified ND | |  | | 8 | 0.925 | 0.23 |  |  |  |  |  |
|  |  |  | Radiation plus modified ND | |  |  | 8 | 0.913 | 0.18 |  |  |  |  |  |
|  |  |  | Radiation | |  |  | 8 | 0.875 | 0.44 |  |  |  |  |  |
|  |  |  | Radical ND | |  |  | 8 | 0.763 | 1.03 |  |  |  |  |  |
|  |  |  | Radiation plus radical ND | |  |  | 8 | 0.675 | 1.3 |  |  |  |  |  |
| Kent (2015) [59] | SF-6D/VR-6D | Patients after a diagnosis of oral cavity or pharyngeal cancer | | |  | | 580 | 0.69 |  |  | 0.68-0.70 |  |  |  |
| Llewellyn-Thomas (1993) [45] | TTO | Larynx cancer patients eligible for a standard four-week RT regimen | | Mild* | Mild** | Time 1 | 24 | 0.721 | 26.2 |  |  |  |  |  |
|  |  |  |  |  |  | Time 2 |  | 0.735 | 23.5 |  |  |  |  |  |
|  |  |  |  |  | Moderate | Time 1 | 36 | 0.750 | 19.9 |  |  |  |  |  |
|  |  |  |  |  |  | Time 2 |  | 0.757 | 19.0 |  |  |  |  |  |
|  |  |  |  |  | Severe | Time 1 | 6 | 0.750 | 24.1 |  |  |  |  |  |
|  |  |  |  |  |  | Time 2 |  | 0.866 | 7.5 |  |  |  |  |  |
|  |  |  |  | Moderate | Mild | Time 1 | 24 | 0.629 | 26.9 |  |  |  |  |  |
|  |  |  |  |  |  | Time 2 |  | 0.571 | 26.4 |  |  |  |  |  |
|  |  |  |  |  | Moderate | Time 1 | 36 | 0.644 | 22.9 |  |  |  |  |  |
|  |  |  |  |  |  | Time 2 |  | 0.667 | 21.8 |  |  |  |  |  |
|  |  |  |  |  | Severe | Time 1 | 6 | 0.700 | 27.0 |  |  |  |  |  |
|  |  |  |  |  |  | Time 2 |  | 0.758 | 15.0 |  |  |  |  |  |
|  |  |  |  | Severe | Mild | Time 1 | 24 | 0.352 | 28.3 |  |  |  |  |  |
|  |  |  |  |  |  | Time 2 |  | 0.429 | 29.2 |  |  |  |  |  |
|  |  |  |  |  | Moderate | Time 1 | 36 | 0.344 | 25.3 |  |  |  |  |  |
|  |  |  |  |  |  | Time 2 |  | 0.381 | 26.7 |  |  |  |  |  |
|  |  |  |  |  | Severe | Time 1 | 6 | 0.233 | 22.7 |  |  |  |  |  |
|  |  |  |  |  |  | Time 2 |  | 0.408 | 39.0 |  |  |  |  |  |
|  | VAS |  |  | Mild | Mild | Time 1 | 24 | 0.826 | 9.8 |  |  |  |  |  |
|  |  |  |  |  |  | Time 2 |  | 0.793 | 13.3 |  |  |  |  |  |
|  |  |  |  |  | Moderate | Time 1 | 36 | 0.793 | 16.0 |  |  |  |  |  |
|  |  |  |  |  |  | Time 2 |  | 0.744 | 13.7 |  |  |  |  |  |
|  |  |  |  |  | Severe | Time 1 | 6 | 0.775 | 13.9 |  |  |  |  |  |
|  |  |  |  |  |  | Time 2 |  | 0.783 | 10.0 |  |  |  |  |  |
|  |  |  |  | Moderate | Mild | Time 1 | 24 | 0.598 | 16.6 |  |  |  |  |  |
|  |  |  |  |  |  | Time 2 |  | 0.623 | 17.8 |  |  |  |  |  |
|  |  |  |  |  | Moderate | Time 1 | 36 | 0.559 | 20.9 |  |  |  |  |  |
|  |  |  |  |  |  | Time 2 |  | 0.532 | 17.8 |  |  |  |  |  |
|  |  |  |  |  | Severe | Time 1 | 6 | 0.647 | 17.4 |  |  |  |  |  |
|  |  |  |  |  |  | Time 2 |  | 0.615 | 14.0 |  |  |  |  |  |
|  |  |  |  | Severe | Mild | Time 1 | 24 | 0.266 | 24.3 |  |  |  |  |  |
|  |  |  |  |  |  | Time 2 |  | 0.276 | 25.3 |  |  |  |  |  |
|  |  |  |  |  | Moderate | Time 1 | 36 | 0.226 | 21.4 |  |  |  |  |  |
|  |  |  |  |  |  | Time 2 |  | 0.209 | 19.2 |  |  |  |  |  |
|  |  |  |  |  | Severe | Time 1 | 6 | 0.128 | 17.6 |  |  |  |  |  |
|  |  |  |  |  |  | Time 2 |  | 0.292 | 31.8 |  |  |  |  |  |
| Loimu (2015) [57] | 15D | Newly diagnosed patients scheduled for receiving CRT | | | Baseline | | 64 | 0.886 | 0.10 |  |  |  |  |  |
|  |  |  |  |  | 3 months | | 54 | 0.829 | 0.12 |  |  |  |  |  |
|  |  |  |  |  | 6 months | | 61 | 0.860 | 0.12 |  |  |  |  |  |
|  |  |  |  |  | 12 months | | 64 | 0.862 | 0.14 |  |  |  |  |  |
| Marcellusi (2015) [36] | TTO | Patients with a confirmed diagnosis of HNC and time from (medical or surgical) treatment no longer than 20 months | | | All patients | | 79 | 0.69 | 0.30 |  | 0.62-0.75 |  |  |  |
|  |  |  |  |  | Males | | 62 | 0.70 | 0.32 |  | 0.62-0.78 |  |  |  |
|  |  |  |  |  | Females | | 17 | 0.64 | 0.21 |  | 0.54-0.74 |  |  |  |
|  | EQ-5D |  |  |  | All patients | | 79 | 0.80 | 0.20 |  |  |  |  |  |
|  |  |  |  |  | Males | | 62 | 0.80 | 0.20 |  |  |  |  |  |
|  |  |  |  |  | Females | | 17 | 0.70 | 0.20 |  |  |  |  |  |
| Noel (2015) [37] | SG | Patients with a minimum of three months after completion of treatment (surgery or RT) and no evidence of recurrent disease | | | All patients | | 100 | 0.91 | 0.17 |  |  |  | 0.2-1.0 |  |
|  |  |  |  |  | Primary surgery | | 54 | 0.93 | 0.17 |  |  |  |  |  |
|  |  |  |  |  | Salvage surgery | | 5 | 0.98 | 0.04 |  |  |  |  |  |
|  |  |  |  |  | Chemotherapy | | 13 | 0.92 | 0.10 |  |  |  |  |  |
|  |  |  |  |  | No chemotherapy | | 87 | 0.91 | 0.18 |  |  |  |  |  |
|  |  |  |  |  | Stage T1 or T2 | | 47 | 0.95 | 0.13 |  |  |  |  |  |
|  |  |  |  |  | Stage T3 or T4 | | 20 | 0.87 | 0.22 |  |  |  |  |  |
|  |  |  |  |  | Tracheotomy and/or feeding tube | | 6 | 0.99 | 0.02 |  |  |  |  |  |
|  |  |  |  |  | No tracheotomy and/or feeding tube | | 94 | 0.91 | 0.17 |  |  |  |  |  |
|  | TTO |  |  |  | All patients | | 100 | 0.94 | 0.14 |  |  |  | 0.3-1.0 |  |
|  |  |  |  |  | Primary surgery | | 54 | 0.95 | 0.13 |  |  |  |  |  |
|  |  |  |  |  | Salvage surgery | | 5 | 0.98 | 0.04 |  |  |  |  |  |
|  |  |  |  |  | Chemotherapy | | 13 | 0.99 | 0.03 |  |  |  |  |  |
|  |  |  |  |  | No chemotherapy | | 87 | 0.94 | 0.14 |  |  |  |  |  |
|  |  |  |  |  | Stage T1 or T2 | | 47 | 0.96 | 0.09 |  |  |  |  |  |
|  |  |  |  |  | Stage T3 or T4 | | 20 | 0.88 | 0.21 |  |  |  |  |  |
|  |  |  |  |  | Tracheotomy and/or feeding tube | | 6 | 0.91 | 0.12 |  |  |  |  |  |
|  |  |  |  |  | No tracheotomy and/or feeding tube | | 94 | 0.95 | 0.14 |  |  |  |  |  |
|  | VAS |  |  |  | All patients | | 100 | 0.76 | 0.19 |  |  |  | 0.2-1.0 |  |
|  |  |  |  |  | Primary surgery | | 54 | 0.76 | 0.20 |  |  |  |  |  |
|  |  |  |  |  | Salvage surgery | | 5 | 0.48 | 0.13 |  |  |  |  |  |
|  |  |  |  |  | Chemotherapy | | 13 | 0.66 | 0.19 |  |  |  |  |  |
|  |  |  |  |  | No chemotherapy | | 87 | 0.77 | 0.18 |  |  |  |  |  |
|  |  |  |  |  | Stage T1 or T2 | | 47 | 0.77 | 0.18 |  |  |  |  |  |
|  |  |  |  |  | Stage T3 or T4 | | 20 | 0.70 | 0.20 |  |  |  |  |  |
|  |  |  |  |  | Tracheotomy and/or feeding tube | | 6 | 0.69 | 0.23 |  |  |  |  |  |
|  |  |  |  |  | No tracheotomy and/or feeding tube | | 94 | 0.76 | 0.19 |  |  |  |  |  |
|  | EQ-5D |  |  |  | All patients | | 100 | 0.82 | 0.18 |  |  |  | -0.07; 1.0 |  |
|  |  |  |  |  | Primary surgery | | 54 | 0.83 | 0.19 |  |  |  |  |  |
|  |  |  |  |  | Salvage surgery | | 5 | 0.62 | 0.17 |  |  |  |  |  |
|  |  |  |  |  | Chemotherapy | | 13 | 0.76 | 0.17 |  |  |  |  |  |
|  |  |  |  |  | No chemotherapy | | 87 | 0.83 | 0.18 |  |  |  |  |  |
|  |  |  |  |  | Stage T1 or T2 | | 47 | 0.83 | 0.18 |  |  |  |  |  |
|  |  |  |  |  | Stage T3 or T4 | | 20 | 0.83 | 0.09 |  |  |  |  |  |
|  |  |  |  |  | Tracheotomy and/or feeding tube | | 6 | 0.78 | 0.14 |  |  |  |  |  |
|  |  |  |  |  | No tracheotomy and/or feeding tube | | 94 | 0.82 | 0.18 |  |  |  |  |  |
|  | HUI3 |  |  |  | All patients | | 100 | 0.75 | 0.25 |  |  |  | -0.06; 1.0 |  |
|  |  |  |  |  | Primary surgery | | 54 | 0.78 | 0.22 |  |  |  |  |  |
|  |  |  |  |  | Salvage surgery | | 5 | 0.37 | 0.29 |  |  |  |  |  |
|  |  |  |  |  | Chemotherapy | | 13 | 0.57 | 0.38 |  |  |  |  |  |
|  |  |  |  |  | No chemotherapy | | 87 | 0.78 | 0.21 |  |  |  |  |  |
|  |  |  |  |  | Stage T1 or T2 | | 47 | 0.80 | 0.21 |  |  |  |  |  |
|  |  |  |  |  | Stage T3 or T4 | | 20 | 0.74 | 0.21 |  |  |  |  |  |
|  |  |  |  |  | Tracheotomy and/or feeding tube | | 6 | 0.73 | 0.25 |  |  |  |  |  |
|  |  |  |  |  | No tracheotomy and/or feeding tube | | 94 | 0.75 | 0.29 |  |  |  |  |  |
|  |  |  |  |  | Larynx | | 17 | 0.59 |  |  |  |  |  |  |
|  |  |  |  |  | Oropharynx | | 14 | 0.76 |  |  |  |  |  |  |
|  |  |  |  |  | Oral cavity | | 67 | 0.78 |  |  |  |  |  |  |
| Outtassi (2016) [48] | EQ-5D | Patients with a confirmed diagnosis of HNC | | |  | | 120 | 0.49 | 0.35 |  |  |  |  |  |
| Parrilla (2015) [49] | EQ-5D | Patients laryngectomized with a stable pulmonary situation with a minimum of 3 months after treatment | | | Baseline (no HME) | | 30 | 0.84 | 0.14 |  |  |  | 0.44-1.00 |  |
|  |  |  |  |  | Week 2 (HME) | | 30 | 0.90 | 0.10 |  |  |  | 0.67-1.00 |  |
|  |  |  |  |  | Week 6 (HME) | | 30 | 0.93 | 0.09 |  |  |  | 0.68-1.00 |  |
|  |  |  |  |  | Week 12 (HME) | | 30 | 0.96 | 0.10 |  |  |  | 0.66-1.00 |  |
| Parthan (2009) [61] | Mapping | Patients with locally advanced inoperable HNC | | | Stable | |  | 0.70 |  |  |  |  | 0.63-0.77* |  |
|  |  |  |  |  | Progressive | |  | 0.67 |  |  |  |  | 0.60-0.74 |  |
|  |  |  |  |  | Response | |  | 0.79 |  |  |  |  | 0.71-0.87 |  |
| Pickard (2016) [52] | EQ-5D (US tariff) | Patients with advanced HNC after at least two cycles of CT | | |  | | 50 | 0.76 | 0.15 |  |  |  |  |  |
| Pottel (2015) [55] | EQ-5D (Belgian tariff) | Patients aged ≥65 years, eligible for curative primary or adjuvant RT | | | Baseline (before treatment) | All | 81 |  |  |  |  | 0.66 |  | 0.55-0.76 |
|  |  |  |  |  |  | Fit* | - |  |  |  |  | 0.76 |  | 0.66-0.76 |
|  |  |  |  |  |  | Vulnerable | - |  |  |  |  | 0.63 |  | 0.29-0.73 |
|  |  |  |  |  | Week 4 (mid-therapy) | All | 81 |  |  |  |  | 0.42 |  | 0.26-0.73 |
|  |  |  |  |  |  | Fit* | - |  |  |  |  | 0.66 |  | 0.39-0.76 |
|  |  |  |  |  |  | Vulnerable | - |  |  |  |  | 0.39 |  | 0.21-0.67 |
|  |  |  |  |  | 2 months (end of treatment) | All | 81 |  |  |  |  | 0.66 |  | 0.29-0.76 |
|  |  |  |  |  |  | Fit* | - |  |  |  |  | 0.74 |  | 0.66-0.76 |
|  |  |  |  |  |  | Vulnerable | - |  |  |  |  | 0.58 |  | 0.23-0.73 |
|  |  |  |  |  | 5 months (follow-up) | All | 81 |  |  |  |  | 0.66 |  | 0.27-0.76 |
|  |  |  |  |  |  | Fit* | - |  |  |  |  | 0.76 |  | 0.66-1.00 |
|  |  |  |  |  |  | Vulnerable | - |  |  |  |  | 0.66 |  | 0.19-0.76 |
|  |  |  |  |  | 12 months | All | 81 |  |  |  |  | 0.64 |  | 0.00-0.76 |
|  |  |  |  |  |  | Fit* | - |  |  |  |  | 0.76 |  | 0.64-1.00 |
|  |  |  |  |  |  | Vulnerable | - |  |  |  |  | 0.57 |  | 0.00-0.74 |
|  |  |  |  |  | 24 months | All | 81 |  |  |  |  | 0.29 |  | 0.00-0.76 |
|  |  |  |  |  |  | Fit* | - |  |  |  |  | 0.76 |  | 0.32-1.00 |
|  |  |  |  |  |  | Vulnerable | - |  |  |  |  | 0.00 |  | 0.00-0.66 |
|  |  |  |  |  | 36 months | All | 81 |  |  |  |  | 0.00 |  | 0.00-0.67 |
|  |  |  |  |  |  | Fit* | - |  |  |  |  | 0.66 |  | 0.00-1.00 |
|  |  |  |  |  |  | Vulnerable | - |  |  |  |  | 0.00 |  | 0.00-0.58 |
| Ramaekers (2011) [51] | EQ-5D (UK tariff) | Patients with a follow-up of at least 6 months after curative RT without evidence of recurrent disease | | | All patients | | 396 | 0.850 | 0.18 |  |  | - |  | - |
|  |  |  |  |  | X0-D0 | | 84 | 0.909 | 0.161 |  |  | 1.000 |  | 0.186 |
|  |  |  |  |  | X0-D1 | | 18 | 0.841 | 0.144 |  |  | 0.796 |  | 0.275 |
|  |  |  |  |  | X1-D0 | | 92 | 0.898 | 0.138 |  |  | 1.000 |  | 0.204 |
|  |  |  |  |  | X1-D1 | | 68 | 0.829 | 0.175 |  |  | 0.814 |  | 0.275 |
|  |  |  |  |  | X1-D2 | | 14 | 0.803 | 0.136 |  |  | 0.796 |  | 0.133 |
|  |  |  |  |  | X2-D0 | | 15 | 0.846 | 0.177 |  |  | 0.850 |  | 0.275 |
|  |  |  |  |  | X2-D1 | | 31 | 0.817 | 0.187 |  |  | 0.812 |  | 0.309 |
|  |  |  |  |  | X2-D2 | | 40 | 0.763 | 0.213 |  |  | 0.778 |  | 0.311 |
|  |  |  |  |  | X2-D3(+) | | 16 | 0.758 | 0.234 |  |  | 0.796 |  | 0.363 |
| Ringash (2000) [42] | TTO | Irradiated laryngeal cancer patients who completed treatment at least 6 months before | | | All | | 112 | 0.914 | 0.156 |  |  |  | 0.25; 1.0 |  |
|  |  |  |  |  | Group 1 | | 84 | 0.878 | 0.174 |  |  |  | 0.25; 1.0 |  |
| Rogers (2006) [50] | EQ-5D (UK tariff) | Patients without evidence of disease after primary surgery for oral/oropharyngeal cancer | | |  | | 224 | 0.75 |  | 0.02 |  |  | -0.18; 1.0 |  |
| Szabo (2012) [20] | SG | Eight vignettes representing disease’s characteristics and ten describing treatment-related toxicities | | | Locoregional (larynx) | | 101 | 0.62 |  | 0.02 | 0.57-0.67 | 0.65 | 0.00-0.98 | 0.50-0.80 |
|  |  |  |  |  | Locoregional (not larynx) | | 101 | 0.61 |  | 0.02 | 0.56-0.66 | 0.63 | 0.03-0.98 | 0.50-0.78 |
|  |  |  |  |  | Recurrent (not larynx) | | 101 | 0.57 |  | 0.02 | 0.52-0.62 | 0.58 | 0.03-0.98 | 0.50-0.78 |
|  |  |  |  |  | Recurrent (larynx) | | 101 | 0.56 |  | 0.02 | 0.51-0.61 | 0.55 | 0.00-0.98 | 0.45-0.73 |
|  |  |  |  |  | Metastatic (not larynx) | | 101 | 0.52 |  | 0.02 | 0.47-0.57 | 0.50 | 0.00-0.98 | 0.38-0.68 |
|  |  |  |  |  | Metastatic (larynx) | | 101 | 0.50 |  | 0.02 | 0.45-0.55 | 0.50 | 0.00-0.98 | 0.35-0.65 |
|  |  |  |  |  | Anemia grade III/IV | | 49 | 0.47 |  | 0.03 | 0.40-0.54 | 0.50 | 0.00-0.98 | 0.30-0.65 |
|  |  |  |  |  | Hematological grade III/IV | | 49 | 0.46 |  | 0.04 | 0.39-0.53 | 0.50 | 0.00-0.98 | 0.33-0.65 |
|  |  |  |  |  | Skin reactions grade I/II | | 52 | 0.45 |  | 0.04 | 0.37-0.52 | 0.50 | 0.03-0.98 | 0.24-0.58 |
|  |  |  |  |  | Peripheral neuropathy grade III/IV | | 49 | 0.44 |  | 0.04 | 0.36-0.51 | 0.45 | 0.00-0.98 | 0.20-0.60 |
|  |  |  |  |  | Treatment cessation due to grade III/IV toxicity | | 52 | 0.44 |  | 0.03 | 0.36-0.51 | 0.50 | 0.03-0.98 | 0.23-0.55 |
|  |  |  |  |  | Nausea/vomiting grade III/IV | | 49 | 0.43 |  | 0.04 | 0.35-0.50 | 0.45 | 0.00-0.98 | 0.25-0.60 |
|  |  |  |  |  | Mucositis/stomatitis grade III/IV | | 49 | 0.43 |  | 0.04 | 0.35-0.50 | 0.45 | 0.00-0.98 | 0.23-0.58 |
|  |  |  |  |  | Anorexia/weight loss grade III/IV | | 52 | 0.40 |  | 0.04 | 0.33-0.47 | 0.48 | 0.00-0.98 | 0.18-0.50 |
|  |  |  |  |  | Skin reactions grade III/IV | | 52 | 0.37 |  | 0.04 | 0.30-0.44 | 0.43 | 0.00-0.98 | 0.14-0.50 |
|  |  |  |  |  | Post-progression (not larynx) | | 101 | 0.34 |  | 0.02 | 0.29-0.39 | 0.38 | 0.00-0.98 | 0.08-0.50 |
|  |  |  |  |  | Post-progression (larynx) | | 101 | 0.34 |  | 0.02 | 0.29-0.39 | 0.38 | 0.00-0.98 | 0.08-0.50 |
|  |  |  |  |  | Hospitalization for grade III/IV toxicity | | 52 | 0.33 |  | 0.04 | 0.26-0.40 | 0.36 | 0.00-0.98 | 0.05-0.50 |
| Truong (2016) [53] | EQ-5D (US tariff) | Untreated stage III or IV cancer patients enrolled on a RCT comparing radiation-cisplatin without cetuximab (CIS) or with cetuximab (CET/CIS) | | | CIS | Baseline | 366 | 0.78 | 0.18 |  |  | 0.82 | 0.17-1.00 | 0.77-0.84 |
|  |  |  |  |  |  | 3 months | - | 0.78 | 0.18 |  |  | - | - | - |
|  |  |  |  |  |  | 12 months | - | 0.84 | 0.17 |  |  | - | - | - |
|  |  |  |  |  | CET/CIS | Baseline | 349 | 0.80 | 0.17 |  |  | 0.83 | 0.20-1.00 | 0.77-0.84 |
|  |  |  |  |  |  | 3 months | - | 0.77 | 0.15 |  |  | - | - | - |
|  |  |  |  |  |  | 12 months | - | 0.84 | 0.16 |  |  | - | - | - |
| van der Donk (1995) [46] | TTO | Scenarios describing the health state of patients treated for T3 laryngeal cancer (*state scenarios*) | RT | | Laryngeal cancer | | 10 | 0.70 |  |  |  |  |  |  |
|  |  |  |  |  | FOM cancer | | 10 | 0.72 |  |  |  |  |  |  |
|  |  |  |  |  | Healthy subjects | | 10 | 0.90 |  |  |  |  |  |  |
|  |  |  |  |  | Clinical experts | | 9 | 0.81 |  |  |  |  |  |  |
|  |  |  | Surgery | | Laryngeal cancer | | 10 | 0.65 |  |  |  |  |  |  |
|  |  |  |  |  | FOM cancer | | 10 | 0.64 |  |  |  |  |  |  |
|  |  |  |  |  | Healthy subjects | | 10 | 0.77 |  |  |  |  |  |  |
|  |  |  |  |  | Clinical experts | | 9 | 0.71 |  |  |  |  |  |  |
|  | SG |  | RT | | Laryngeal cancer | | 10 | 0.61 |  |  |  |  |  |  |
|  |  |  |  |  | FOM cancer | | 10 | 0.83 |  |  |  |  |  |  |
|  |  |  |  |  | Healthy subjects | | 10 | 0.84 |  |  |  |  |  |  |
|  |  |  |  |  | Clinical experts | | 9 | 0.92 |  |  |  |  |  |  |
|  |  |  | Surgery | | Laryngeal cancer | | 10 | 0.62 |  |  |  |  |  |  |
|  |  |  |  |  | FOM cancer | | 10 | 0.63 |  |  |  |  |  |  |
|  |  |  |  |  | Healthy subjects | | 10 | 0.68 |  |  |  |  |  |  |
|  |  |  |  |  | Clinical experts | | 9 | 0.84 |  |  |  |  |  |  |
|  | RS |  | RT | | Laryngeal cancer | | 10 | 0.66 |  |  |  |  |  |  |
|  |  |  |  |  | FOM cancer | | 10 | 0.78 |  |  |  |  |  |  |
|  |  |  |  |  | Healthy subjects | | 10 | 0.68 |  |  |  |  |  |  |
|  |  |  |  |  | Clinical experts | | 9 | 0.78 |  |  |  |  |  |  |
|  |  |  | Surgery | | Laryngeal cancer | | 10 | 0.45 |  |  |  |  |  |  |
|  |  |  |  |  | FOM cancer | | 10 | 0.50 |  |  |  |  |  |  |
|  |  |  |  |  | Healthy subjects | | 10 | 0.47 |  |  |  |  |  |  |
|  |  |  |  |  | Clinical experts | | 9 | 0.57 |  |  |  |  |  |  |
|  | TTO | Scenarios describing the health state of patients treated for T3 laryngeal cancer including temporary and permanent side effects, life expectancy, tumor recurrence rates, and probability of treatment outcomes (*dynamic scenarios*) | RT | | Laryngeal cancer | | 10 | 0.66 |  |  |  |  |  |  |
|  |  |  |  |  | FOM cancer | | 10 | 0.62 |  |  |  |  |  |  |
|  |  |  |  |  | Healthy subjects | | 10 | 0.73 |  |  |  |  |  |  |
|  |  |  |  |  | Clinical experts | | 9 | 0.80 |  |  |  |  |  |  |
|  |  |  | Surgery | | Laryngeal cancer | | 10 | 0.62 |  |  |  |  |  |  |
|  |  |  |  |  | FOM cancer | | 10 | 0.61 |  |  |  |  |  |  |
|  |  |  |  |  | Healthy subjects | | 10 | 0.66 |  |  |  |  |  |  |
|  |  |  |  |  | Clinical experts | | 9 | 0.73 |  |  |  |  |  |  |
|  | SG |  | RT | | Laryngeal cancer | | 10 | 0.65 |  |  |  |  |  |  |
|  |  |  |  |  | FOM cancer | | 10 | 0.70 |  |  |  |  |  |  |
|  |  |  |  |  | Healthy subjects | | 10 | 0.83 |  |  |  |  |  |  |
|  |  |  |  |  | Clinical experts | | 9 | 0.91 |  |  |  |  |  |  |
|  |  |  | Surgery | | Laryngeal cancer | | 10 | 0.71 |  |  |  |  |  |  |
|  |  |  |  |  | FOM cancer | | 10 | 0.66 |  |  |  |  |  |  |
|  |  |  |  |  | Healthy subjects | | 10 | 0.76 |  |  |  |  |  |  |
|  |  |  |  |  | Clinical experts | | 9 | 0.85 |  |  |  |  |  |  |
|  | RS (VAS) |  | RT | | Laryngeal cancer | | 10 | 0.60 |  |  |  |  |  |  |
|  |  |  |  |  | FOM cancer | | 10 | 0.63 |  |  |  |  |  |  |
|  |  |  |  |  | Healthy subjects | | 10 | 0.64 |  |  |  |  |  |  |
|  |  |  |  |  | Clinical experts | | 9 | 0.69 |  |  |  |  |  |  |
|  |  |  | Surgery | | Laryngeal cancer | | 10 | 0.49 |  |  |  |  |  |  |
|  |  |  |  |  | FOM cancer | | 10 | 0.55 |  |  |  |  |  |  |
|  |  |  |  |  | Healthy subjects | | 10 | 0.52 |  |  |  |  |  |  |
|  |  |  |  |  | Clinical experts | | 9 | 0.55 |  |  |  |  |  |  |
| Weiss (1994) [43] | TTO | Stage N0 patients free of disease after different treatment options | Observation | |  | | 3 | 1.0 |  |  |  |  |  |  |
|  |  |  | Neck dissection | |  |  | 3 | 0.97 |  |  |  |  |  |  |
|  |  |  | Radiotherapy | |  |  | 3 | 0.97 |  |  |  |  |  |  |
|  |  |  | Salvage surgery (successful) | |  |  | 3 | 0.94 |  |  |  |  |  |  |
| Yong (2012) [62] | Mapping | Patients with early stage cancer | IMRT | | Immediately after treatment | |  | 0.810 |  |  |  |  |  |  |
|  |  |  |  |  | 6 months | |  | 0.853 |  |  |  |  |  |  |
|  |  |  |  |  | 12 months | |  | 0.925 |  |  |  |  |  |  |
|  |  |  | 3DCRT | | Immediately after treatment | |  | 0.810 |  |  |  |  |  |  |
|  |  |  |  |  | 6 months | |  | 0.853 |  |  |  |  |  |  |
|  |  |  |  |  | 12 months | |  | 0.868 |  |  |  |  |  |  |

*Abbreviations:*

3DCRT: three dimensional conformal radiotherapy; CI: confidence interval; CO2: transoral CO2 laser excision; CRT: chemoradiotherapy; CT: chemotherapy; D: dysphagia; FOM: floor-of-the-mouth; HME: heat and moisture exchanger; HSUV: health state utility value; HUI3: Health Utility Index Mark 3; IMRT: intensity-modulated radiation therapy; IQR: interquartile range; MRND: modified radical neck dissection; NA: not available; ND: neck dissection; PEG: gastrostomy tube; RCT: randomized controlled trial; RS: rating scale; RT: radiotherapy; SE: standard error; SD: standard deviation; SG: standard gamble; SLNB: sentinel lymph node biopsy; SOHND: supraomohyoid neck dissection; TL: total laryngectomy; TORS: transoral robotic surgery; TTO: time trade-off; VAS: visual analogue scale; WW: watchful waiting; X: xerostomia; XRT: external radiation therapy.

*Notes:*

**Govers (2016)**

Group 1: patients without previous mucosal malignancies, local recurrences or second primary tumors in the head and neck cancer region.

Group 2: patients without previous mucosal malignancies, local recurrences, or second primary tumors and without adjuvant (chemo)radiotherapy in the head and neck cancer region.

**Hamilton (2016)**

Group 1: participants ranking CRT first in a previous exercise.

Group 2: participants ranking TL first in a previous exercise.

**Llewellyn-Thomas (1993)**

Mild/Moderate/Severe*: health state scenarios describing increasing levels of three radiation-induced disorders (i.e. mouth/throat pain, fatigue, inability to converse)

Mild/Moderate/Severe**: end-of-therapy groups as self-determined by the patients

Time 1/Time 2: outset/end of therapy

HSUVs were converted on a 0-1 scale (from a 0-100 one).

**Parthan (2009)**

* Uncertainty range: 2.5 percentile and 97.5 percentile.

**Pottel (2015)**

Fit/vulnerable patients: classification based on geriatric-8 (G-8) assessment at baseline

**Ringash (2000)**

Group 1: excluding patients who claimed they had or did not want perfect health.
